# ST-Elevation Myocardial Infarction as a Late Complication of Mild Coronavirus Disease 2019 Infection: A Case Report

**DOI:** 10.7759/cureus.21943

**Published:** 2022-02-05

**Authors:** Mahmoud Abdelnabi, Juthipong Benjanuwattra, Abdallah Almaghraby, Yehia Saleh, Kenneth Nugent

**Affiliations:** 1 Cardiology and Angiology Unit, Clinical and Experimental Internal Medicine Department, Medical Research Institute, Alexandria University, Alexandria, EGY; 2 Internal Medicine Department, Texas Tech University Health Sciences Center, Lubbock, USA; 3 Cardiology Department, Faculty of Medicine, Alexandria University, Alexandria, EGY; 4 Cardiology Department, Houston Methodist DeBakey Heart & Vascular Center, Houston, USA

**Keywords:** percutaneous coronary intervention, vasculitis, myocardial infarction, sars-cov-2, covid-19

## Abstract

Endothelial dysfunction with subsequent thrombosis and, less commonly, vasculitis has been implicated during the active phase of severe acute respiratory syndrome coronavirus 2 (SARS-COV-2) infection. However, less has been described during the recovery phase or as late sequelae. Here, we report a case of acute anterior wall ST-elevation myocardial infarction in a female patient with no medical history of cardiovascular risk factors as a post-infectious complication of coronavirus disease 2019 (COVID-19). Coronary angiography revealed total occlusion of her left anterior descending, right coronary arteries, and tight stenosis in the left circumflex artery. Successful revascularization with a staged percutaneous coronary intervention was achieved. To date, there is not much data regarding the late cardiovascular sequelae of COVID-19 and its possible mechanisms. Prolonged follow-up, even for mild cases of COVID-19, is advised for early diagnosis and treatment of long-term complications of COVID-19.

## Introduction

Coronavirus disease 2019 (COVID-19)-mediated vasculopathy has been increasingly recognized as a serious complication of severe acute respiratory syndrome coronavirus 2 (SARS-CoV-2) infection. The complex interplay between endothelial dysfunction from direct viral infection and systemic inflammation is associated with various thrombotic complications and vasculitides [1,2]. Despite the resolution of the primary infection, vascular complications, including stroke, ST-elevation myocardial infarction (STEMI), and vasculitides, in various sites have been reported in a subset of patients [3-6].

## Case presentation

We present the case of a 58-year-old female with an unremarkable medical history except for a resolved COVID-19 infection. Three months ago, she had experienced fever, shortness of breath, and labile blood pressure, and had tested positive for COVID-19 by real-time reverse transcriptase-polymerase chain reaction. She did not require supplemental oxygen or hospitalization and was managed conservatively at home. Afterward, she started to complain of progressive exertional chest pain and dyspnea grade II relieved by rest; nevertheless, she did not seek medical advice. She presented three months after her initial diagnosis with COVID-19 to our medical facility complaining of acute onset of retrosternal chest pain radiating to her left arm and associated with diaphoresis and sweating. Her physical examination was unremarkable apart from tachycardia (heart rate of 105 beats/minute). Electrocardiography revealed anterolateral ST-segment elevations. She was diagnosed with anterior STEMI and transferred immediately for coronary angiography (CA). CA revealed total thrombotic occlusion of the left anterior descending artery (LAD) and total occlusion of the right coronary artery (RCA) receiving retrograde collaterals from the left system, and a proximal tight lesion in the left circumflex artery (LCx) (Video 1).

**Video 1:** CA showing proximal total thrombotic occlusion of LAD, proximal total occlusion of RCA, which is receiving retrograde collaterals from the left system, and proximal tight lesion of the LCx. CA: coronary angiography; LAD: left anterior descending artery; RCA: right coronary artery; LCx: left circumflex artery

After patient counseling, the decision was made to proceed with total revascularization with staged percutaneous coronary intervention (PCI). She underwent successful primary LAD PCI (Video 2).

**Video 2:** CA showing successful PCI of LAD using one stent with TIMI II flow. CA: coronary angiography; PCI: percutaneous coronary intervention; LAD: left anterior descending artery; TIMI: Thrombolysis in Myocardial Infarction

After uneventful observation in the cardiac care unit for 48 hours, she underwent successful PCI of LCx and RCA (Video 3).

**Video 3:** CA showing patent LAD stent, and successful PCI of the LCx using one stent and RCA using two stents with TIMI III flow in all vessels. CA: coronary angiography; LAD: left anterior descending artery; PCI: percutaneous coronary intervention; LCx: left circumflex artery; RCA: right coronary artery; TIMI: Thrombolysis in Myocardial Infarction

She was discharged on dual antiplatelet therapy with close follow-up visits.

## Discussion

The pathogenesis of SARS-COV-2-mediated vasculopathy has been proposed as follows: (1) Co-expression of the angiotensin-converting enzyme 2 receptor and transmembrane serine protease 2 in endothelial cells facilitates the viral entry, thereby promoting endothelial damage, inflammation, and prothrombotic state [7,8]. The infected endothelial cells then lose their ability to produce nitric oxide, an essential regulator involved in many physiological processes, including suppression of leukocyte migration, platelet adhesion, and inflammation. (2) An indirect endothelial injury aggravated by an exaggerated inflammatory response and complement activation. Postmortem analysis of a series of patients with COVID-19 infection revealed evidence of endotheliitis and inflammatory cell infiltration across the vascular bed in various organs, including the heart [9]. A few case reports have shown that patients with resolved COVID-19 continue to be susceptible to thromboembolic complications, such as myocardial infarction and stroke during both immediate [5] and subacute post-infectious periods [4]. A 64-year-old woman with previous mildly symptomatic COVID-19 infection suffered an acute ischemic stroke and silent myocardial infarction five weeks after COVID-19 infection, albeit inflammatory markers were not elevated [4].

In addition to thrombotic complications, there is scarce evidence showing that coronary artery vasculitis can also manifest as a post-infectious complication of COVID-19, despite having mild or no previous pulmonary infection, as a part of the multisystem inflammatory syndrome [10,11] Given that our patient presented with isolated coronary artery vasculitis, without other systemic manifestations, or elevated inflammatory markers, a wide range of heterogeneity in the clinical presentation is suggested. COVID-19 vasculopathy has been implicated in vessels of all sizes, including giant cell arteritis, as reported in the case of a 47-year-old woman who presented with temporal headache and jaw claudication four months after COVID-19 infection [3].

To date, the mechanisms responsible for the association between COVID-19 and coronary artery vasculopathy, after the hyperinflammatory state has subsided, have not been determined. Several questions remain unanswered, including “how can we stratify those who are at higher risk of developing vasculopathy after resolution of COVID-19, and how long will they be susceptible to those complications?.” Long-term close follow-up of even mild cases of COVID-19 is required to diagnose late cardiovascular complications. Two-dimensional speckle-tracking echocardiography with left ventricular global longitudinal strain can be used as a validated method to determine early or late myocardial dysfunction following COVID-19 infection [12].

## Conclusions

So far, data regarding late cardiovascular complications of COVID-19 and its possible mechanisms are not available. Follow-up is required for even mild cases of COVID-19 for the early diagnosis and treatment of long-term consequences of COVID-19.
